# Effects of polydopamine coatings on nucleation modes of surface mineralization from simulated body fluid

**DOI:** 10.1038/s41598-020-71900-3

**Published:** 2020-09-11

**Authors:** Giovannimaria Murari, Nathalie Bock, Huan Zhou, Lei Yang, Teresa Liew, Kate Fox, Phong A. Tran

**Affiliations:** 1 Queensland University of Technology (QUT), 2 George Street, Brisbane, QLD Australia; 2 Centre in Regenerative Medicine, QUT, Brisbane, Australia; 3 Translational Research Institute (TRI), Brisbane, QLD Australia; 4 Faculty of Health, School of Biomedical Sciences, QUT, Australian Prostate Cancer Research Centre (APCRC-Q), Brisbane, QLD Australia; 5 Center for Health Science and Engineering, Tianjin Key Laboratory of Materials Laminating Fabrication and Interface Control Technology, School of Materials Science and Engineering, Hebei University of Technology, Tianjin, 300130 China; 6 School of Medicine, The University of Queensland, Brisbane, Australia; 7 Center for Additive Manufacturing and School of Engineering, RMIT University, Melbourne, VIC 3000 Australia; 8 Interface Science and Materials Engineering Group, School of Chemistry, Physics and Mechanical Engineering, QUT, Brisbane, Australia

**Keywords:** Materials science, Biomaterials, Materials for devices, Soft materials

## Abstract

Polydopamine (PDA) has been recently used as a versatile priming layer for further functionalization of a biomaterial surface, particularly in biomimetic mineralization of biomaterials. Yet most of the existing literature is on inorganic substrates and the underlying effects of the PDA layer coatings on the nucleation and mineralization process and the mineral-substrate interface have not been clearly identified. Here we aimed to investigate the effects of the PDA layer on the nucleation and growth and interfacial morphology of calcium phosphate mineral layer (CaP) from 10× simulated body fluid (10× SBF) on polymeric substrates. It is found that the nucleation of CaP on PDA-coated surface favors a mixed “islanding” and planar growth mode (Stranski–Krastanov) while the “islanding” mode (Volmer–Weber) was observed on the surface without PDA. This different early nucleation stage of mineralization was found to correlate with a more “bonded” interface between the mineral layer and the PDA-coated substrates, a slight increase in the interfacial strength and a different delamination mode. This study therefore provided new insights on how polydopamine priming layer influenced the mineralization process and the interface between the mineral layer and the substrate.

## Introduction

Polydopamine (PDA) has recently been used as a versatile functionalization tool that could be applied to a range of substrates to improve wettability, biocompatibility and to allow for further bio-functionalization^1,2^. One of the advantages of PDA priming is the easiness of application: PDA can been readily deposited under slightly basic conditions (pH ~ 8.5) by the oxidative polymerization of dopamine, to form a biocompatible layer that irreversibly adheres to the surface of a number of materials^3–9^. PDA has been used as an agent to induce deposition of calcium phosphate mineral on various substrate materials as a biomimetic coating.

Investigation into the early phase of mineralization on PDA-coated surfaces has been lacking. The main contribution of PDA to the mineralization of calcium phosphate (CaP) layers is from its surface charged groups, such as catechol or amine groups which can have strong interaction with calcium and phosphate ions^4,5^. Yet the majority of existing work in this area only focused on the structure and properties of the complete mineral layers. For example, PDA was used to induce the formation a mix CaP-Ag coating on titanium nanotubes, yet the authors focused solely on characterizing the morphology and microstructure of the coating and its antibacterial and osteointegration properties^10^. PDA was also mixed with calcium phosphate cement and the mixture was shown to promote mineralization from concentrated SBF solution^11^. This latter study demonstrated that CaP formed through co-precipitation with PDA, yet the understanding of how PDA influences this precipitation was lacking. Given that the charged groups on PDA could interact strongly with the ions from the mineralizing solution, it is of great interest to investigate the early phase of precipitation/mineralization and correlate the findings with the interface properties between the mineral layer and the substrate.

Mineralization started with nucleation on a substrate from the oversaturated solution containing calcium and phosphate ions, such as simulated body fluid and the nuclei continued to grow to eventually completely cover the substrate^12^. This nucleation-growth process can occur in one of the following 3 modes depending on the substrate-coating adhesion and coating cohesion energies^13–18^. For a coating that presents a coating-substrate adhesion energy significantly stronger than coating-coating cohesion energy, a planar growth mode (Frank–van der Merwe mode ) is dominant where a planar layer expands to cover the substrate. Conversely, when cohesion is significantly stronger island-forming mode (“islanding”, Volmer–Weber) occurs predominantly where isolated clusters grow in size to cover the substrate. A mixture of islanding and planar mode (Stranski–Krastanov mode) is also common in many systems. Experimentally, coating formation on hydrophobic surfaces occurs predominantly through “islands” whereas mixed islanding and planar modes (known as Stranski–Krastanov modes) occur on more hydrophilic surfaces^13,15,16^.

In this study, polycaprolactone (PCL) was used as a model polymeric substrate to investigate specifically the effects of PDA functionalization on the early phase of mineralization and its effects on the interface between the substrate and the coating. PCL was chosen because of its extensive investigation for bone tissue engineering or medical devices, yet it lacks intrinsic osteoconductivity thus CaP coating on PCL has been used to improve its bioactivity^19–21^. PCL is also slightly hydrophobic thus surface etching (in strong alkali solutions) or plasma treatment has been often performed before immersing in mineralizing solutions^21–24^. PDA has been used to enhance mineralization on PCL^25,26^ yet its effects on the early phase of mineralization and the coating-substrate interface remains to be investigated.

## Materials and methods

Medical grade polycaprolactone (Purasorb PC12, Purac, Mw of 120 kDa) used was from Corbion Purac. Dopamine hydrochloride, DMEM, FBS and P/S were from Sigma Aldrich. Reagents to make 10× SBF (including NaCl, KCl, CaCl_2_.2H_2_O, MgCl_2_.6H_2_O, Na_2_HPO_4_, NaHCO_3_) were from Chem-Supply (Australia). PCL films were prepared by solvent casting. The casting solution was prepared dissolving PCL in chloroform to obtain 10% w/v, then casted in glass petri dishes and dried overnight in a fume hood to form PCL film of approximately 200–300 µm thick. For plasma treatment, the films were placed in a plasma chamber (Plasma Cleaner, PDC-002-HP, Harrick Plasma) using the following parameters: O_2_ (flow rate of 7.9 mL/min) and Ar (flow rate of 7.3 mL/min) at pressure of 200 mTorr for a 15 min. The plasma treated sample is referred hereafter to as pPCL.

For PDA functionalization of pPCL, we used a solution of 1 mg/ml dopamine hydrochloride in carbonate buffer at pH 8.5 according to previous work^5^. The pPCL films were immersed in that solution in a Petri dish placed on an orbital shaker (at a speed of 30 rounds per minute) for different periods of time.

The morphology of PCL, pPCL, and PDA-functionalized pPCL was evaluated using atomic force microscopy (AFM, NT-MDT solver SPM with silicon AFM probes Tap 300 from Ted Pella). The instrument was placed on an active vibration isolation Halcyonics i4 (Accurion). Scans were taken with a frequency of 1 Hz in semi-contact mode and with scan size ranging from 5 × 5 µm to 40 × 40 µm.

Contact angle measurement was performed on a FTA200 (Ten Angstroms) to determine the wettability of samples. A droplet of 8 µL of MilliQ water was placed onto the tested specimens and contact angle was measured within 30 s.

For CaP deposition on polymeric surfaces, a coating process based on simulated body fluid is commonly used because of a low processing temperature and ability to form uniform coatings on the surface of complex 3D geometries^27^. The term SBF was introduced by Kokubo et al*.*, as a solution with ionic concentration and pH close to that of human blood plasma^28,29^. Incubating a substrate in SBF can induce the formation of a coating layer of carbonated apatitic calcium phosphate chemically mimicking the composition of the inorganic phase of bone^28–32^. This method is therefore particularly applicable to polymers of low melting temperatures such as PCL, PLGA which are not suitable for traditional CaP coating methods such as plasma (thermal) spraying^33,34^. A formulation of 10× SBF (pH 4.0) containing NaCl (58.43 g/L), KCl (0.373 g/L), CaCl_2_.2H2O (3.675 g/L), MgCl_2_.6H_2_O (1.016 g/L) and Na_2_HPO_4_ (1.42 g/L) was used to accelerate the mineralization^13,24^. The pH of this 10× SBF was adjusted to 6 using NaHCO3 before substrates were placed into the solution and incubated at 37 °C for 5 min (the mineral nucleation stage) and 60 min (the complete mineralization group). Samples were rinsed with MilliQ water and air-dried before analysis.

The cross section of membranes were imaged using a Jeol 1400 TEM microscope after resin embedding and sectioning (using a microtome).

The CaP-coated samples were characterized using scanning electronic microscopy (Zeiss FESEM (Carl Zeiss AG) coupled with an EDX detector). The FTIR analyses were made on a Nicolet iS50 FT-IR, (Thermo scientific). Analysis was performed in Attenuated Total Reflection (ATR) mode with an investigated range 4,000–375 cm^−1^. A KBr beam splitter was used, with 64 scans per sample and a resolution of 4 wavenumbers. X-ray photoelectron spectroscopy (XPS, K-Alpha, Thermo Scientific) was performed to evaluate the elemental composition on the substrate during early mineralization stage. Survey scan was taken at 200 eV, step size 0.1 eV and dwell time 10 ms (3 sweeps); high resolution scans for the individual elements were taken at 50 eV, step size 0.1 eV and dwell time 10 ms (10 sweeps).

The interface strength was evaluated by peel tests^35^. PCL coated samples of size 40 mm × 20 mm were fixed on metal plates by glue and then pressure-sensitive tape (standard ASTM D5486) was pressed with known pressure and a fixed holding time on the coated side of the sample (0.25 Mpa for 30 s). The peeling measurements were performed on a Micro tester 5848, by Instron, with cross-head speed set to 5 mm/s during the tests. After the test, the samples were imaged under SEM, to quantify the coating area that has been delaminated during the test.

The interface strength between CaP layer and substrates was also compared between different groups by sonication. The CaP coated samples were immersed in MilliQ water and sonicated with a SONICLEANTM 250 HD sonicator at an operating power of 120 W for 60 min at 30 °C. After sonication, the supernatants containing the dislodged coating were collected, digested with HNO_3_ 70% w/v and analyzed with ICP-OES (Perkin Elmer 8300DV). The remaining coating on the sonicated samples were determined by digesting the whole samples in HNO_3_ 70% w/v and analyzed with ICP-OES.

Experiments were done with at least 5 replicates unless otherwise indicated. Data were reported as mean ± standard error of the mean (SEM) and statistical difference was analyzed with *t-test*.

## Results and discussion

### Plasma treatment and PDA functionalization of PCL surface

**Figure 1:**
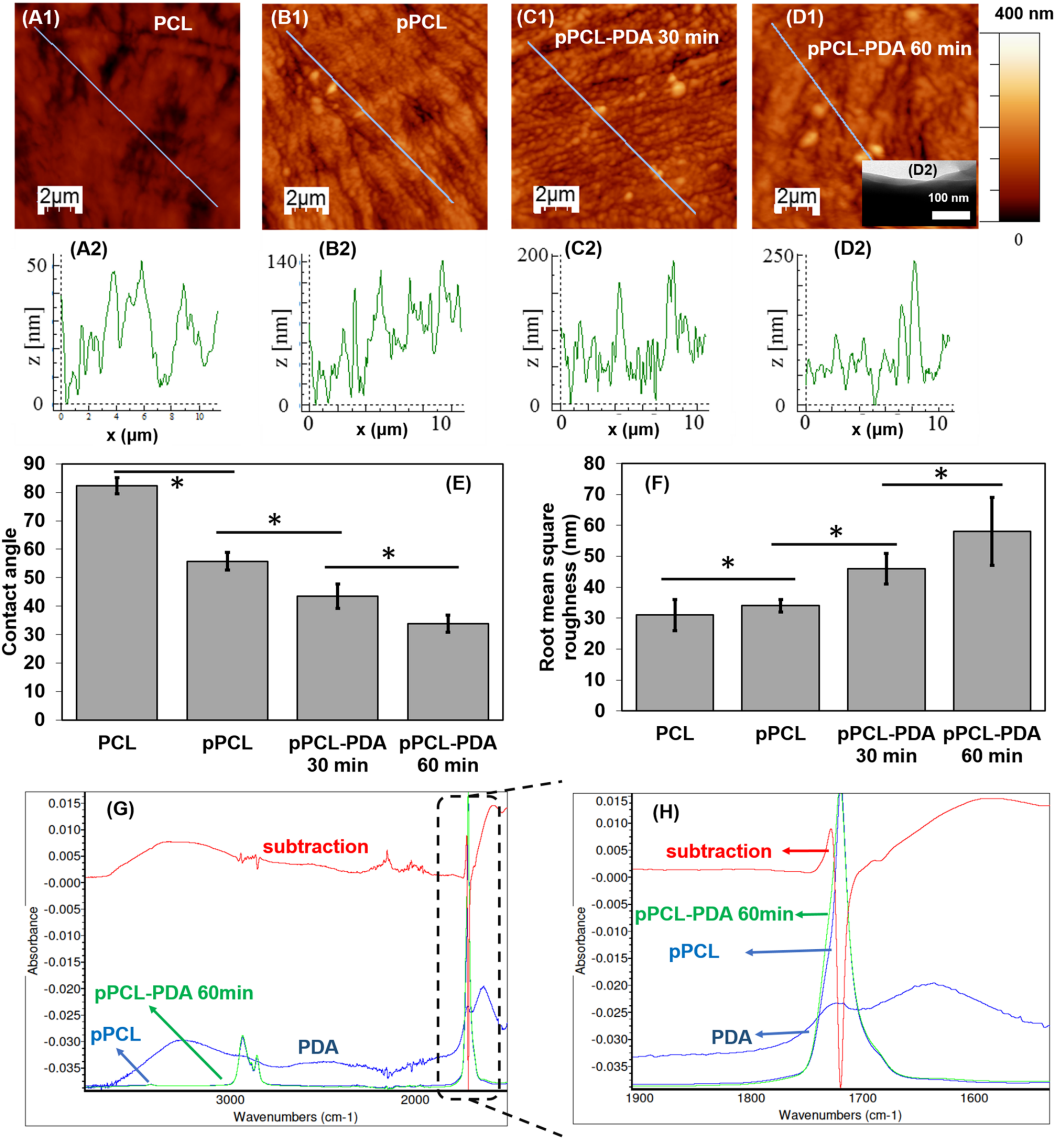
AFM and contact angle analysis of dopamine polymerization on plasma treated PCL. (**A1**–**D1**) Representative AFM micrographs of PCL, plasma treated PCL (pPCL) and pPCL after 30 min and 60 min of dopamine polymerization, respectively. (**A2**–**D2**) Representative line analysis demonstrating the polydopamine on the surface as submicron- and nano-sized clusters. Longer dopamine polymerization time onto PCL increased the surface roughness (**F**) and hydrophilicity (**E**). FTIR analysis comparing PCL and PCL-PDA (60 min of functionalization) showing a small yet clear contribution of C = O stretching in quinone group in PDA (around 1721 cm^−1^) group in PDA. Data = mean ± SEM.

First, the polymerization of dopamine onto plasma treated PCL substrates was studied. Atomic force microscopy (AFM) showed that PDA appeared as nano-and submicron-clusters on the substrate and that surface roughness (Fig. 1A–D and F) increased with increasing polymerization time.

Increasing the PDA coating time from 60 to 120 min did not significantly change the surface roughness or contact angles (RMS = 52 ± 9 nm, contact angle = 41 ± 4 degree), which is in agreement with previous reports^24^. Plasma treatment and PDA immobilization also made the PCL surface more hydrophilic as indicated by the decreasing water contact angles (Fig. 1E). PDA appeared as a thin layer (~ 40 nm after 60 min of functionalization) on PCL substrate and gave rise to a small yet clear increase of absorbance around 1721 cm^−1^ in the FTIR spectrum (Fig. 1G,H). XPS and surface charge analysis (Supplementary Figures S1 and S2) showed a clear evolution of surface functional groups during functionalization and an oxidation-ring closure mechanism was proposed accordingly.

### Effects of PDA functionalization on early stage of CaP mineralization

Since surface roughness and wettability were reported previously as important factors inducing apatite mineralization on surfaces^36–38^, it was hypothesized that PDA functionalization of PCL would have strong effects on the formation of CaP on the PCL surface.

**Figure 2:**
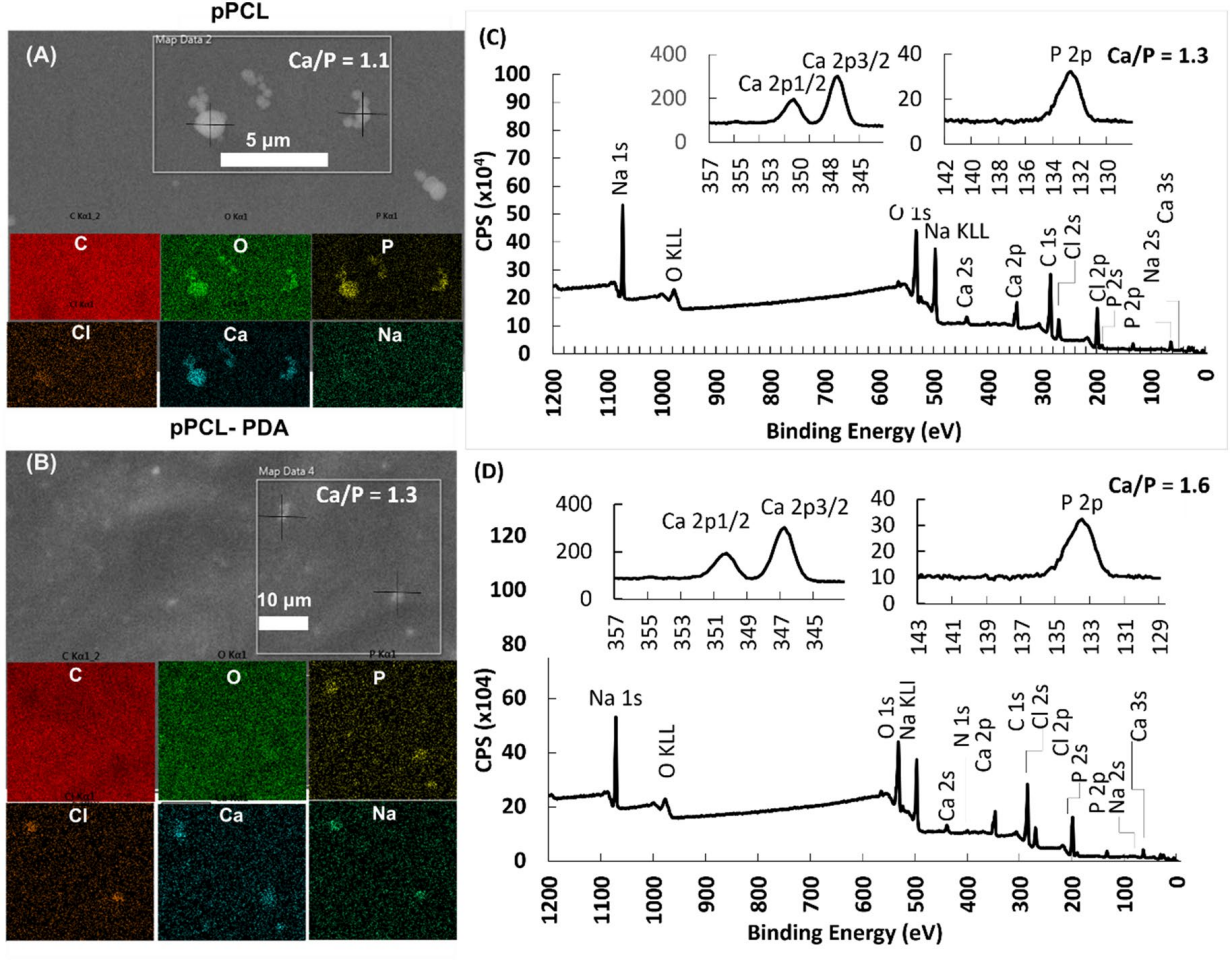
PDA effects on the nucleation during early mineralization. EDX and XPS analysis showed morphology and chemical compositions: (**A**) on pPCL substrates Ca, O, P-rich particulate aggregates exhibited characteristics of strong cohesion (“islanding” or Volmer–Weber growth mode) and (**B**) on pPCL with PDA treatment substrates higher density of P, Cl, Ca, Na-rich single particles suggested strong adhesion to substrate (Stranski growth mode). (**C**,**D**) XPS analysis showed similar oxidation states of early CaP on both substrates, yet higher Ca/P atomic ratios on pPCL-PDA (**D**) than on pPCL (**C**).

The substrates were immersed in 10× SBF solution for 5 min to study the nucleation and growth—early stages of mineralization. The nuclei appeared as spherical micro- and sub-micro sized aggregates on pPCL (Fig. 2A) and as individual smaller particles on pPCL-PDA (Fig. 2B). Spherical clusters on pPCL exhibited characteristics of islanding growth mode indicating a stronger mineral phase cohesion than its adhesion on the substrate^13,18^. Individual particles on pPCL-PDA samples suggested strong mineral-substrate adhesion and the Stranski–Krastanov (islanding-and-planar growth) mode of the coating^13,17^.

Mineralization occurs via 3 major kinetic modes: Volmer–Weber (VM mode) or “islanding” for clusters growth and Stranski–Krastanov (SK) or mixed islanding-and-planar-layer growth^13,17^. In the current study, it was clear that the introduction of PDA favored the SK growth mode in the early stages of coating growth, supported by higher hydrophilicity and the nucleation sites offered by the irreversibly adhered PDA layer^5^. Conversely, CaP formed on plasma-treated PCL favored the VW mode, resulting in layers of strong coating-coating cohesion. XPS was used to analyze the oxidation states and chemical compositions; the results showed similar peak locations yet higher Ca/P atomic ratio on pPCL-PDA (Fig. 2D) than on pPCL (Fig. 2C). This higher Ca/P ratio indicated higher absorption of Ca cations in relation to phosphate anions on the PDA coating.

### PDA functionalization’s effects on the mineral—substrate interface

**Figure 3:**
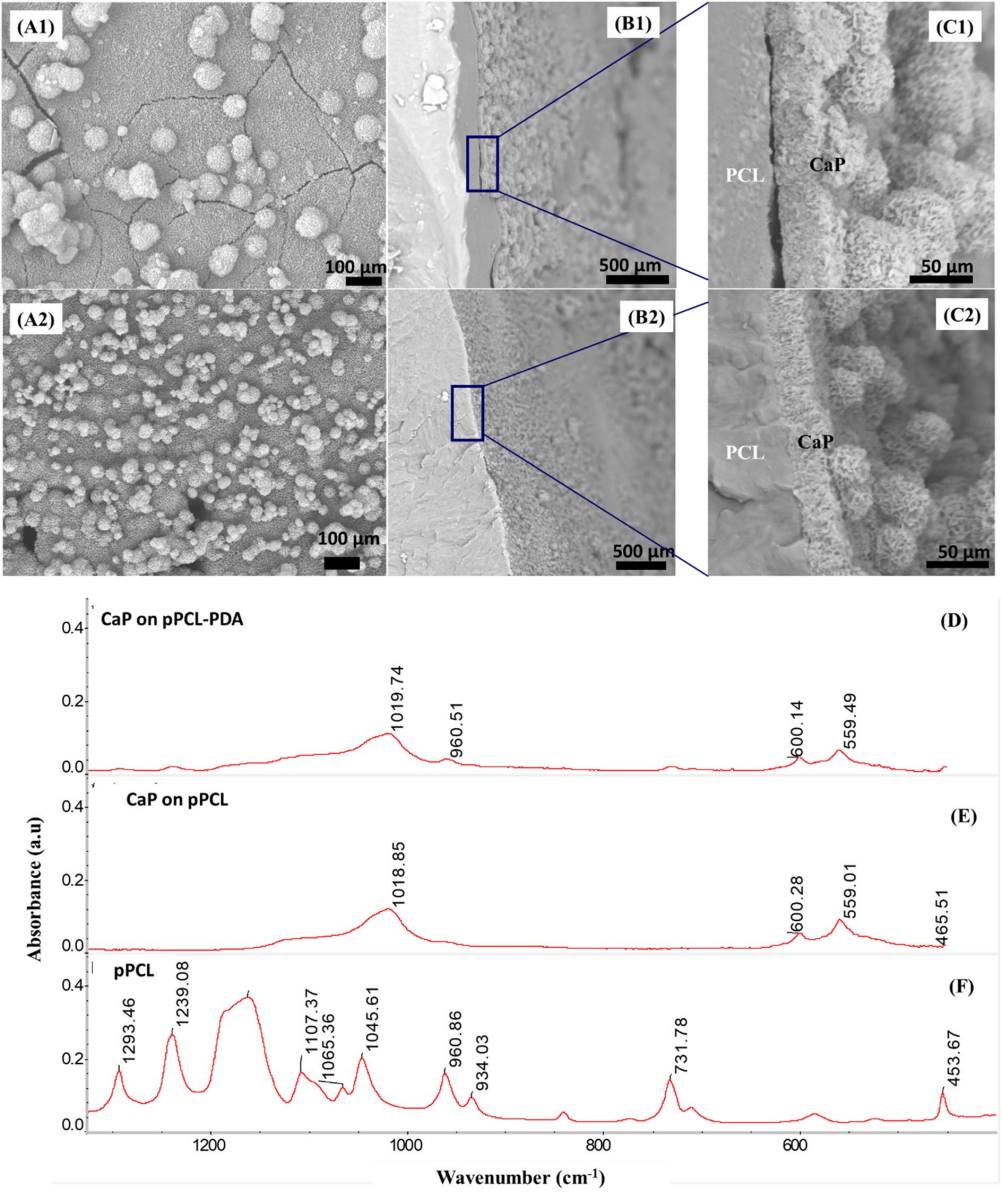
Morphology of complete mineral layers on plasma-treated PCL (**A1**–**B1**–**C1**) and PDA-functionalized PCL (**A2**–**B2**–**C2**) and FTIR analysis (**D**–**F**). Layers on plasma-treated PCL exhibited extensive cracking and delamination while plasma-treated PCL with PDA treatment displayed no cracking, and tight interface with the substrate.

The difference in nucleation and growth modes during early stage of mineralization was expected to result in significant difference in the interface between the substrate and the mineral layer at the later stage. Figure 3 shows that the CaP coating on plasma-treated PCL (pPCL) exhibited extensive cracking and some between the substrate and the mineral layer. On the other hand, the CaP coating on PDA functionalized surface was crack-free and bonded to the substrate.

FTIR analysis (Fig. 3) showed no significant differences between the mineral formed on pPCL and PDA-pPCL. For the pPCL samples, the peak at 1,160 cm^−1^ corresponds to C–O and C=C stretching in the amorphous phase; at 1,239–1,240 cm^−1^ corresponding to asymmetric COC stretching; 1,293 cm^−1^ to C–O and C=C stretching in the crystalline phase^39^. In CaP-coated pPCL and pPCL-PDA, peaks at 1,018–1,019 cm^−1^ corresponds to ν3 PO_4_^3−^ and those at 559,600 cm^−1^ corresponds to ν4 PO_4_^3−^^13,40^. Since PDA was irreversibly immobilized on surfaces^6–9^, it is proposed here that PDA only modified the mineralization process in the early stages (i.e., the first layer of CaP coating). This explained the FTIR results which showed no significant difference between CaP coatings on pPCL and pPCL-PDA (Fig. 3) as in attenuated total reflection (ATR)—FTIR, the evanescent wave (or the penetration depth of the IR radiation) exists in only ~ 1 µm thick top layer.

**Figure 4:**
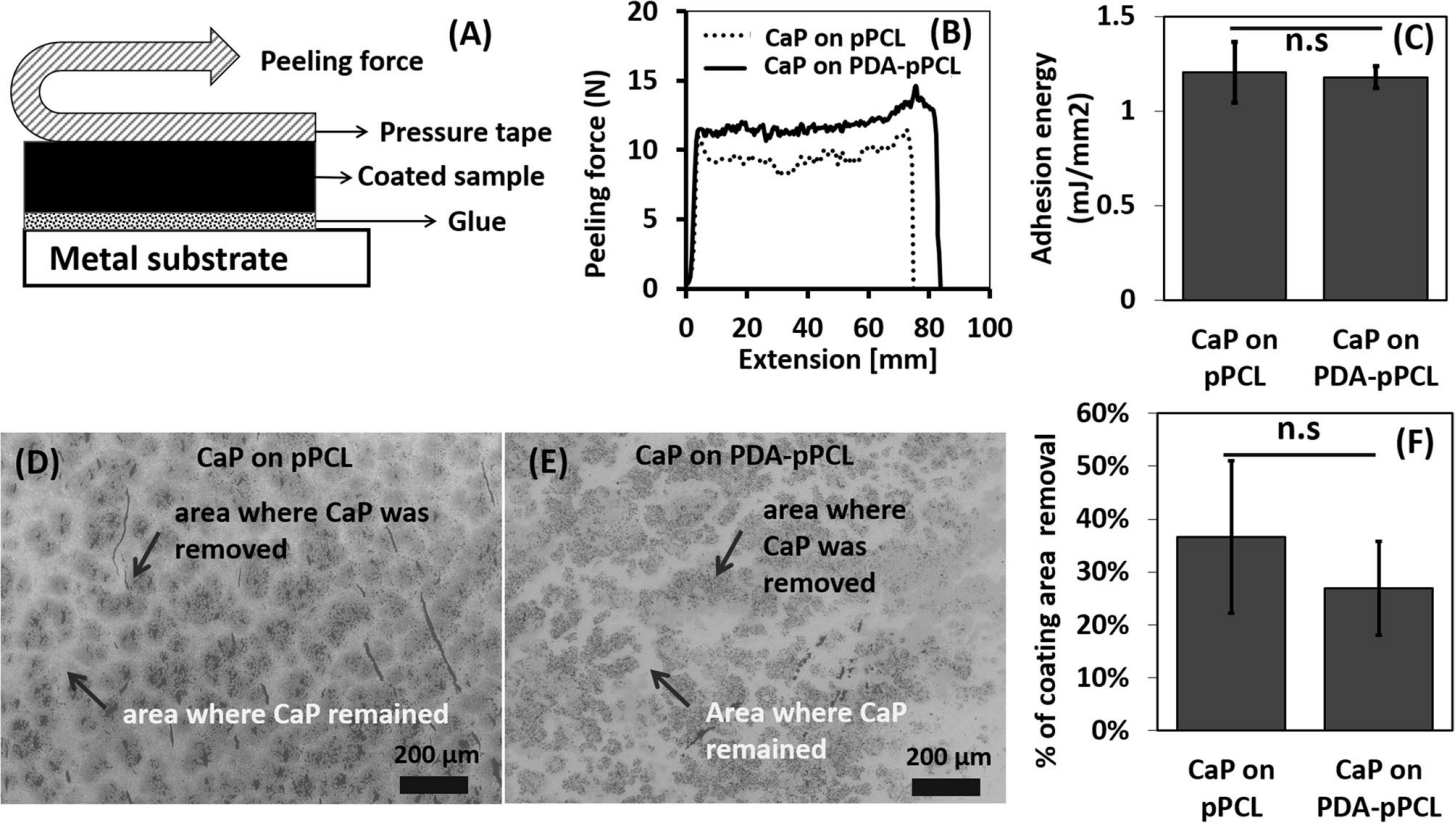
Mineral layers formed on PDA coated PCL showed slightly higher peeling resistance (less coating removed) than that on plasma treated PCL without PDA. (**A**) schematic of peel test. (**B**) representative force-extension curves. (**C**) Quantification of adhesion energy by image contrast analyses. (**D**,**E**) Representative SEM images of surface after peel test. (**F**) Quantification of percentage of coating removal (using image processing of the back-scattered electron (BSE) images based on the contrast of CaP (bright areas) and bare substrate (dark areas)).

The interface strength of mineral-substrate was investigated using peel-tests. The samples were glued to a metal substrate and a pressure tape was pressed against the mineral layer and the tape was pulled (Fig. 4A). The peeling force and extension were recorded and adhesion energy was calculated from the area laying under the force-extension curves (Fig. 4B,C). No significant difference was found in the adhesion energy between the CaP on pPCL and CaP on PDA-pPCL.

The substrates were imaged with a SEM after the peel tests and the percentage of coating area that had been removed during the tests was determined by analyzing the images. The back-scattered electron gave a clear contrast between CaP (bright) and the bare polymer substrate (dark) and allowed for quantification of area coverage (Fig. 4D, E). The percentage of coating removal in terms of area was found slightly lower (without significant difference) on the PDA-coated group (Fig. 4F).

Peel tests have been commonly used to study bond strength but have also been shown to be not sensitive enough for studying mineral layers on polymer substrates as reported by other research groups that studied calcium phosphate mineralization onto poly(carbonate urethane)^41,42^. The interface strength of mineral-substrate was then investigated using an ultrasound test where the samples were immersed in water and exposed to an ultrasound. The samples were then imaged with SEM to study the interface microscopically. The amount of mineral dislodged into the solution was determined using ICP-OES.

**Figure 5:**
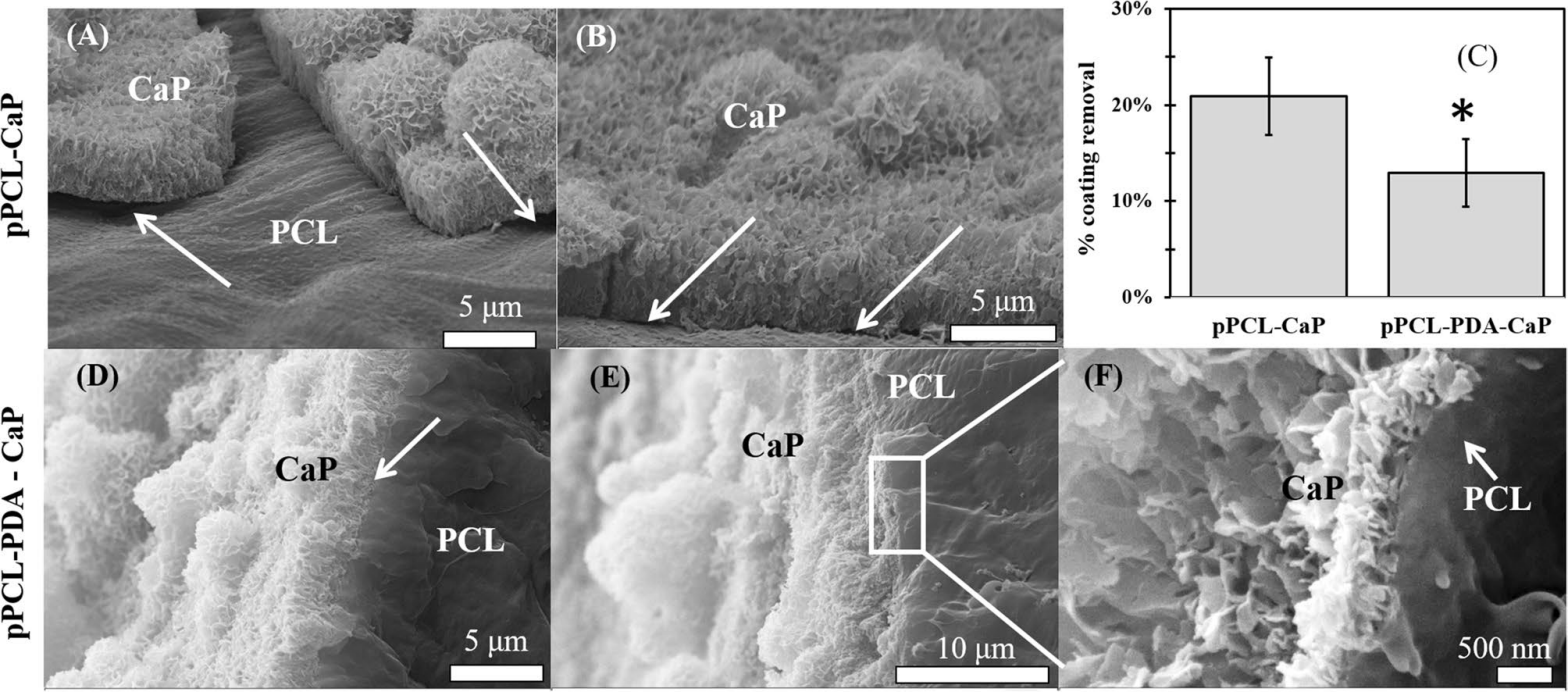
Mineral layer on PCL-PDA was found more resistant to delamination during sonication tests. (**A**,**B**) Delamination of the mineral layers formed on plasma treated PCL clearly indicated adhesion-failure mode. (**C**) Percentage of coating delamination determined by measuring Ca in the dislodged mineral and the remained mineral (using ICP-OES). (Data = mean ± SE, n = 6). (**D**,**E**) Mineral layer on PCL-PDA retained the strong CaP-substrate bond and only the top layer was partially removed suggesting a cohesion-failure mode (**F**).

The mineral layers demonstrated different modes of detachment. On pPCL the coating-substrate interface was clearly disrupted, and the delamination occurred at this interface (Fig. 5A,B). On pPCL-PDA, the interface appeared to be intact (Fig. 5D,E) and the delamination destroyed the CaP-CaP interface (Fig. 5F). The percentage of coating delamination was found to be significantly lower on PDA-pPCL samples (Fig. 5C).

Extensively used as a technique to remove surface contaminants or loosely bound particles, ultra-sonication can be used to apply high sound pressure on samples and allow for direct comparison of coating delamination between samples and has been successfully used for studying coating attachment^43,44^. In the current study, ultra-sonication method was able to show a slightly higher resistance to delamination of the mineral layers on pPCL-PDA group than on the pPCL group.

## Conclusion

In this study, the effects of PDA functionalization on the mineralization of calcium phosphate from supersaturated solution on a model polymeric substrate PCL were investigated. The PDA functionalization of PCL substrate increased surface roughness and hydrophilicity. Mineralization on plasma treated PCL (without PDA surface functionalization) exhibited Volmer–Weber (islanding) growth mode and a gap at the mineral layer-substrate interface. In contrast, the nucleation and growth of mineral layer on PDA functionalized surface appeared to occur mainly via Stranski–Krastanov (islanding & planar growth) mode and formed crack-free coating layer with a tight interface with the underlying substrate. The mineral layer on PDA-functionalized substrate was also found to have slightly stronger interface strength. Together, the findings of this study provided further insights into the influence of PDA functionalization on the formation and interface properties of calcium phosphate mineral layer on polymer substrates.

## Supplementary information

Supplementary information
