# Supplementary material for: Effects of polydopamine coatings on nucleation modes of surface mineralization from simulated body fluid
Source: Sci Rep. 2020 Sep 11;10:14982. doi: 10.1038/s41598-020-71900-3 (PMC7486375; doi:10.1038/s41598-020-71900-3)
Supplement: Supplementary file 1 — Supplementary information [file 41598_2020_71900_MOESM1_ESM.docx]

**Effects of Polydopamine Coatings on Nucleation Modes of Surface Mineralization from Simulated Body Fluid**

Giovannimaria Murari^a^, Nathalie Bock^a,b,c,d^ Huan Zhou^e^, Yang Lei^e^, Teresa Liew^f^, Kate Fox^g^ and Phong A. Tran^a,b,h,*^

*^a^Queensland University of Technology (QUT), 2 George Street, Brisbane, QLD, Australia*

*^b^Centre in Regenerative Medicine, QUT*

*^c^Translational Research Institute (TRI), Brisbane, QLD, Australia*

*^d^School of Biomedical Sciences, Faculty of Health, QUT and Australian Prostate Cancer Research Centre (APCRC-Q), Brisbane, QLD, Australia*

*^e^Center for Health Science and Engineering, Tianjin Key Laboratory of Materials Laminating Fabrication and Interface Control Technology, School of Materials Science and Engineering, Hebei University of Technology, Tianjin 300130, China.*

*^f^School of Medicine, The University of Queensland, Brisbane, Australia.*

*^g^ Center for Additive Manufacturing & School of Engineering, RMIT University, VIC, 3000 Australia*

*^h^Interface Science and Materials Engineering group, School of Chemistry, Physics and Mechanical Engineering, QUT.*

** corresponding author: phong.tran@qut.edu.au*

**Supplementary data:**


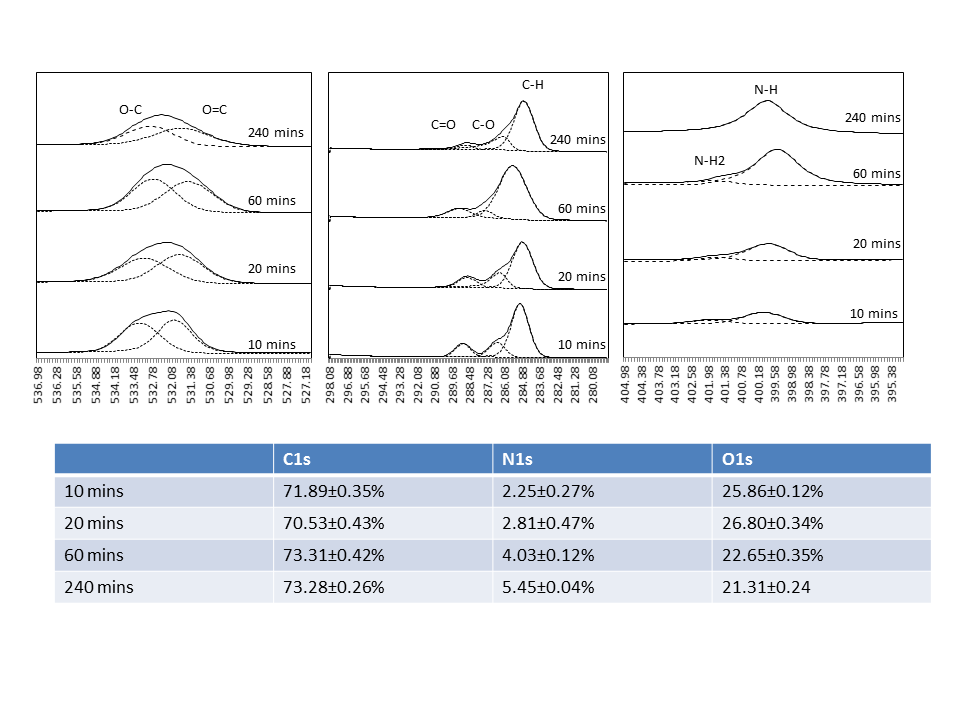


**Figure S1**. XPS investigation of kinetics of dopamine polymerization and immobilization on plasma-treated PCL surfaces. Deconvolution of the peaks showed contributions from different bond components: O1s from 532 eV (O=C) and 533 eV (C-OH); C1s showed contributions from 284.8 eV (C-H), and 287 eV (C-OH) and 289 eV(C=O); and N1s from 399 eV (N-H) and 402 eV (N-H_2_). The atomic percentage of nitrogen deposited on sample surface was 2.25% after 10 minutes, 2.81% after 30 minutes, 4.0% after 60 minutes and 5.45% after 240 minutes, indicating the increasing dopamine polymerization and immobilization on PCL surface. N1s spectra suggested that this increase in N was mostly from the increase in NH groups. The O=C peak was initially higher (at 10 minutes) and gradually decreased relatively to O-C peak. This was also observed in the deconvolution of C1s peaks.


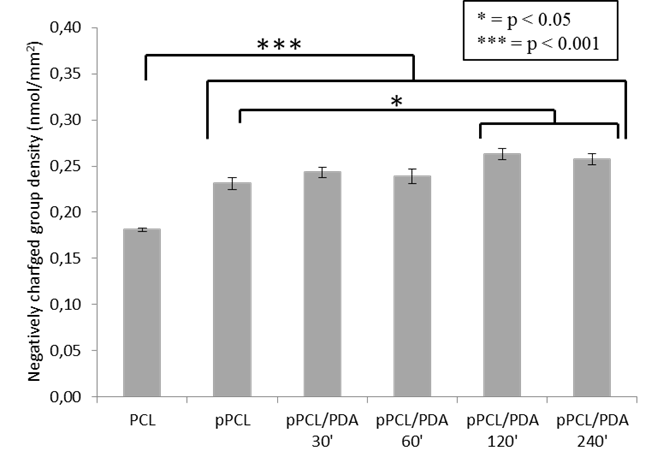


**Figure S2**. Negatively charged group density on PDA-functionalized surface (Data = mean ± SE, n = 5). Colorimetric assays indicated that both the plasma treatment and the PDA deposition significantly increased the negatively charged groups on PCL surface (Figure 3). No statistical difference was found between PDA groups, but the plasma treatment introduced significantly less groups than 120 and 240 min of PDA deposition.

The above XPS analysis and results on the surface charge group measurement revealed important information about the polymerization of dopamine. Despite the fact that PDA has been widely formed on a range of materials as functionalization layers, the polymerization and PDA structures remain to be fully elucidated [1]. It was proposed that dopamine polymerizes through oxidation & ring closure followed by connection of monomer units by C-C bond formation [2]via the benzo moiety or the pyrrole groups [1, 3]. Direct C-C bond formation connecting dopamine was also suggested [4-6]. Concerning the nature of monomers, it has been proposed that they consist of indoles [7] or indole moieties of different oxidation states [8]. PDA structures were also proposed to consist of two fused indole rings as monomer units [1].

In the current study, the N spectra of the samples at time points up to 240 minutes indicated a small fraction of primary amine NH_2_ present during the early phases of polymerization which then disappeared likely due to the cyclization (ring-closure) as the PDA matured [1] (Figure 2C). This also explain the net increase in negative charge density of PCL surface after immobilization of PDA: the slight positive charge of NH was offset by the larger negative charge of catechol groups [9-11]. The relative decrease in C=O groups compared to C-OH groups seen in the XPS deconvolution of O1s and C1s suggested that the dopamine polymerization progressed from the oxidation of dopamine to form dopaminequinone monomer (Figure 4, structure **2**) which then formed the polymer network consisted of moieties of different oxidation states (structures **3-7**) (Figure 4). In the beginning more of **3** and **4** were formed which were then converted to the less oxidized species **5** and **6**, due to the reduced available oxygen in the synthesis solution. A small fraction of 7 was present at the beginning of the reaction (N1s spectra in Figure 2) and completely disappeared at 240 minutes indicating complete ring closure at the later time point.


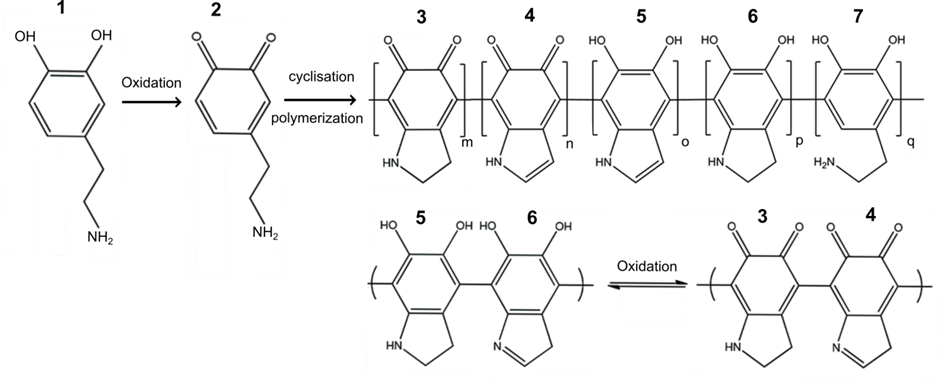


**Figure S3**. Proposed schematic of dopamine polymerization in carbonate buffer at pH 8.5.

1. Liebscher, J.r., et al., *Structure of polydopamine: a never-ending story?* Langmuir, 2013. **29**(33): p. 10539-10548.

2. Yu, F., et al., *Experimental and theoretical analysis of polymerization reaction process on the polydopamine membranes and its corrosion protection properties for 304 Stainless Steel.* Journal of Molecular Structure, 2010. **982**(1-3): p. 152-161.

3. Ye, Q., F. Zhou, and W. Liu, *Bioinspired catecholic chemistry for surface modification.* Chemical Society Reviews, 2011. **40**(7): p. 4244-4258.

4. Mrówczyński, R., et al., *New versatile polydopamine coated functionalized magnetic nanoparticles.* Materials Chemistry and Physics, 2013. **138**(1): p. 295-302.

5. Binns, F., et al., *Studies related to the chemistry of melanins. Part XIII. Studies on the structure of dopamine-melanin.* Journal of the Chemical Society C: Organic, 1970(15): p. 2063-2070.

6. Della Vecchia, N.F., et al., *Building‐block diversity in polydopamine underpins a multifunctional eumelanin‐type platform tunable through a quinone control point.* Advanced Functional Materials, 2013. **23**(10): p. 1331-1340.

7. Lynge, M.E., et al., *Polydopamine—a nature-inspired polymer coating for biomedical science.* Nanoscale, 2011. **3**(12): p. 4916-4928.

8. Si, J. and H. Yang, *Preparation and characterization of bio-compatible Fe3O4@ Polydopamine spheres with core/shell nanostructure.* Materials Chemistry and Physics, 2011. **128**(3): p. 519-524.

9. Yabushita, M., *A Study on Catalytic Conversion of Non-Food Biomass into Chemicals: Fusion of Chemical Sciences and Engineering*. 2016: Springer.

10. Ball, V., *Polydopamine Nanomaterials: Recent Advances in Synthesis Methods and Applications.* Frontiers in bioengineering and biotechnology, 2018. **6**.

11. Park, J., et al., *Polydopamine-based simple and versatile surface modification of polymeric nano drug carriers.* ACS nano, 2014. **8**(4): p. 3347-3356.
